# Ecological interactions in the Scratchpads virtual research environment

**DOI:** 10.3897/BDJ.7.e47043

**Published:** 2019-11-27

**Authors:** Edward Baker, Steen Dupont, Vincent Stuart Smith

**Affiliations:** 1 The Natural History Museum, London, United Kingdom The Natural History Museum London United Kingdom

**Keywords:** ecological informatics, biodiversity informatics, ecological interactions

## Abstract

**Background:**

The Natural History Museum, London has a number of online databases that describe interactions between species, including the HOSTS database of lepidopteran host plants (Robinson et al. 2010) and a database of Dipterocarp Seed Predators. These databases were generally bespoke software, which has increased the technical work necessary to sustain these resources. The decision was taken to migrate these to either the Scratchpads Virtual Research Environment (VRE) (Smith et al. 2011) or to the museum's Data Portal (Scott et al. 2019), depending on the complexity of the existing resource, as both are being sustained by the Informatics Group at the Natural History Museum, London. Resources that can be best represented as a single table were moved to the Data Portal, while those best represented in a relational model were transferred to Scratchpads. In addition, the Phthiraptera.info Scratchpad (Smith and Broom 2019), which already contained ecological interaction data, was migrated to the new system.

**New information:**

This paper describes the implementation within the Scratchpads VRE of a new ecological interactions module that is capable of handling the needs of these projects, while at the same time is flexible to handle the needs of future projects with different data sources.

## Introduction

In order to understand life on Earth, it is essential to understand not only the distribution and traits of species, but how they interact with each other. Biodiversity informatics as a discipline has created global infrastructures for both species distribution (GBIF) and trait (TraitBank) data. Global Biotic Interactions (GloBI: Poelen et al. 2014) is arguably an equivalent infrastructure for ecological interaction datasets.

Ecological interaction datasets are, like natural history specimens, fragmented and widely distributed. They can be found scattered through scientific literature and specimen labels in museums. Numerous publications have synthesised interaction datasets for taxonomic groups (e.g. cockroaches Roth and Willis 1960, Lepidoptera Beccaloni et al. 2008). Widespread adoption of computerised databases and, later, the internet as a dissemination platform, brought these taxon-specific datasets into the digital era (e.g. cestodes Lefebvre et al. 2009). Virtual research environments, such as Scratchpads, provide a community tool for collating disparate data around a specific taxonomic group. This paper describes an extension to the scratchpads platform to facilitate recording of biotic interactions and sharing these data with GloBI.

The need for biodiversity informatics to address species interactions was set as a challenge by Hardisty et al. (2013) and the benefits of digital systems for managing ecological interactions have been demonstrated by Trivellone et al. (2018).

## Project description

### Design description

The existing datasets to be migrated were all of a similar format: two species had been documented interacting in a bibliographic reference, often at a specified location. While this model formed the basis of the implementation, we expanded this model to allow for interactions that are recorded from museum specimens (for an example see Dupont and Baker 2018).

The introduction of non-native species into an ecosystem may result in novel interactions behaviour. For example, the accidental introduction of the stick insect *Carausius morosus* (Sinéty, 1901) into the San Diego area means it has been found on many plants it would not have encountered in its native habitat in southern India (Baker 2015a), including plants that are also not native to San Diego. One potential question that could be answered is "what are the foodplants of Californian stick insects?" In some cases, the exclusion of both non-native stick insects and plants would be desirable in answering this question; the status of both species can therefore be specified (the implementation is aligned to the establishmentMeans property of DarwinCore; Table 1). DarwinCore recommends a controlled vocabulary including the terms used here, but does define a complete controlled vocabulary (https://terms.tdwg.org/wiki/dwc:establishmentMeans). The desirability of a controlled vocabulary has been discussed by Baskauf (2016) and our design allows for such a vocabulary to replace ours when it is available. We do not propose one here because such a vocabulary would have use cases far beyond the scope of this project.

Another example of potentially confusing data for phasmids is the numerous food plants that are successfully used to rear these species in captivity (e.g. Baker 2010). While potentially useful for studies of diet acceptability or breadth (in phasmids: Blüthgen et al. 2005, Junker et al. 2008; in general: Beccaloni and Symons 2000, Symons and Beccaloni 1999), again there are occasions where it would be desirable to exclude such records and the option to separate these records of non-native foodplants is provided (Table 2).

In addition, some interactions have significant importance, such as the defoliation of food and timber crops by stick insects (Baker 2015b). Consequently, the module supports efforts to record the importance of an interaction to one of several values (Table 3). These values are currently not based on a widely used controlled vocabulary, instead being taken from examples within our source datasets.

The migrated hosts data (Robinson et al. 2010) for Lepidoptera and associated host plants have been used in several bodies of work, including the studies of the usability of tortricid moth as a biocontrol agent of ferns in Thailand (Pratt et al. 2016), the general herbivory of conifers in the new world (Brown 2018) and the impact and importance of Geometrid caterpillars as primary biomass consumers in terrestrial ecosystems, such as the Andean fauna (Bodner et al. 2010). A more extensive use of the Robinson et al. (2010) Lepipotera hosts data is a paper on the evolution of the gustatore receptor gene family and the influence of these on host plant adaptation in Nymphalids (Suzuki et al. 2018). The selection of the model species, chosen by Suzuki et al. (2018), was specifically based on *Vanessa cordui* Linnaeus (Lepidopteara: Nymphalidae), because this is recorded as one of the most polyphagous butterfly species.

The above examples are limited to the Phasmida, a small order of insects with less than 4,000 valid species (Brock et al. 2016) and the Lepidoptera, whose host plants have been extensively studied. Despite already requiring a data model beyond 'Species A interacts with Species B according to author C', further exploration of the datasets to be imported revealed additional complications, the most notable of which being the part of the organism involved in interaction. The HOSTS database includes examples where the caterpillars feed on non-leaf parts of plants, including flowers and tubers. In contrast, parasitic louse interactions can be specific, not only to a single species of host, but also to a specific area of that species, such as their wings, head and neck or rump (e.g. Johnson et al. 2012). At present, these are free text fields and not confined to controlled vocabularies, instead using the verbatim data from sources. Controlled vocabularies for anatomy are becoming available, for example Uberon (Mungall et al. 2012) with a focus on vertebrate animals and the Hymenoptera Anatomy Ontology (Deans et al. 2012). It is likely that different Scratchpad communities will want to use different vocabularies and we will monitor developments in this area.

The Phthiraptera Scratchpad (http://phthiraptera.info/) documents approximately 12,000 interactions between parasitic lice (Subinfraorder Phthiraptera) and their mammal and bird hosts. Multiple mammal classifications are used, reflecting the fact that different authorities have used different host classifications when compiling checklists for blood sucking lice (superfamily Anoplura) and chewing lice (superfamilies Amblycera, Ischnocera and Rhynchophthirina). This extensive database underpins a significant body of research on parasitic lice, which is used as a model to study co-speciation.

The Scratchpads project was conceived and developed as part of a much wider portfolio of biodiversity informatics platforms and systems, so from the outset, the system described here was designed to operate with the Global Biotic Interactions project (GloBI; Poelen et al. 2014) via the Natural History Museum's Data Portal (Scott et al. 2019).

### Funding

Funding was provided by the Natural History Museum to employ EB during part of 2016.

## Web location (URIs)

Homepage: http://scratchpads.eu

Bug database: https://github.com/NaturalHistoryMuseum/scratchpads2/issues

## Technical specification

Platform: Drupal; Scratchpads

Programming language: PHP

## Repository

Type: Git

Browse URI: https://github.com/NaturalHistoryMuseum/scratchpads2/tree/master/sites/all/modules/custom/ecoint

## Usage rights

### Use license

Other

### IP rights notes

The code developed for this project is, like the rest of the Scratchpads project, released under the GNU General Public License v2.0. Scratchpad users have fine-grain control over the licence applied to each piece of content on their site, but the project encourages the use of open data licences following Hagedorn et al. (2011).

## Additional information

### Implementation

**Figure 1.**
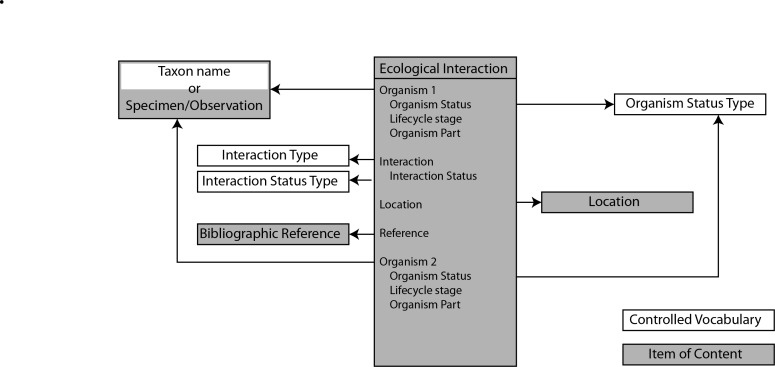
Relationships of the Ecological Interaction content type with other Scratchpad controlled vocabularies and content types. The controlled vocabulary for Organism Status Type is found in Table 1, those for Interaction Status Type in Table 2).

The implementation creates a new content type (Drupal: node type) for ecological interactions. Each interaction (a Drupal node) has a unique URL and identifier (UUID). The UUID uniquely identifies each interaction and should persist if the dataset is aggregated, as a means to trace the provenance of the data in the source dataset (e.g. to correct errors or add annotation). The Scratchpads enivronment has defined content types for bibliographic references, specimens and observations and locations, as well as tools for manipulating biological classification. The new ecological interactions content type links to these existing content types and classifications as shown in Fig. 1.

**Figure 2.**
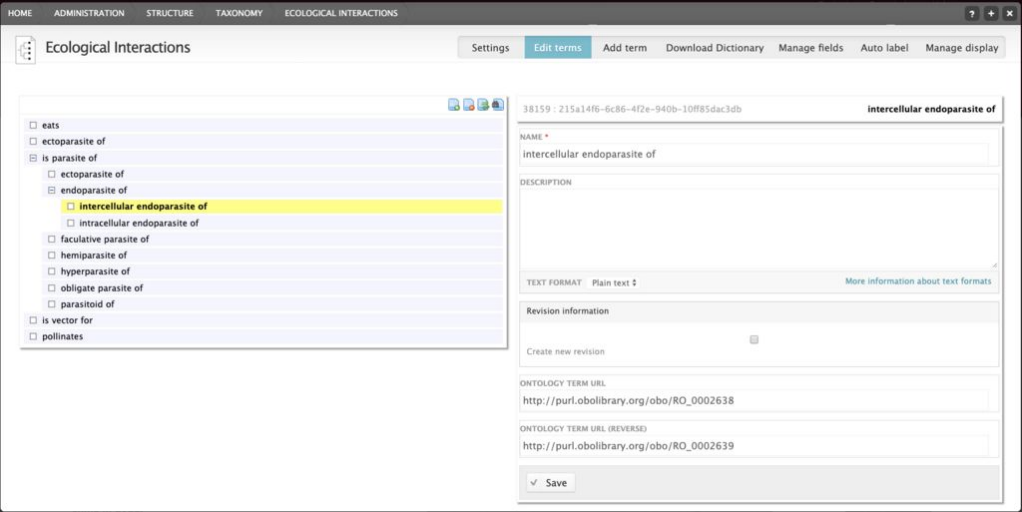
Editing interface for types of ecological interaction.

The Relations Ontology (RO; https://www.ebi.ac.uk/ols/ontologies/ro) provides several ontology terms for describing inter-species interactions, which have been adopted by the module described here. The system presents a human-readable description (e.g. "eats") to the user, but additionally stores the relevant URI from the RO. In addition, the reverse URI is also stored (e.g. "eaten by" is the reverse of "eat"). At present, this functionality is not used, but in the future, this will allow for more advanced searches. These terms are stored in a Drupal taxonomy, allowing terms to be stored in a hierarchy (i.e. "ectoparasite of" is a child term of "parasite of"; Fig. 2).

**Figure 3.**
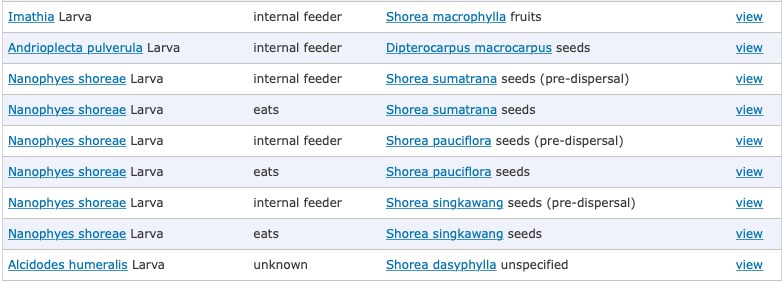
Ecological interactions display page on the Scratchpad *Dipterocarp Seed Predators* (http://dsp.myspecies.info/interactions)

To maintain visual coherence with other Scratchpads features and for increased search speed, the main ecological interactions page (showing all interactions; Fig. 3) makes use of an Apache Solr search backend allowing rapid faceted search of interactions.

A text file suitable for ingest by GloBI is found at /interactions.txt on Scratchpad sites where the module is enabled (e.g. http://phthiraptera.info/interactions.txt). This file can be harvested by external aggregators.

### Future plans

The NHM is undertaking work to combine the output of several Scratchpad sites, as well as other sources, to create an institutional 'interactions bank' that will provide a unified entry point for these disparate interactions datasets. The NHM Data Portal is used by NHM staff to publish their research datasets, whereas Scratchpads can be used by both NHM and external researchers. For this reason, it is currently possible to contribute to GloBI directly from an individual Scratchpad and NHM-affiliated researchers, in future, will be able to contribute via the interactions bank.

An example of a Scratchpad hosted project, that is starting to adopt the ecological interactions module, is the BioAcoustica database (Baker et al. 2015) that is documenting records of acoustically orientating parasites and their hosts (e.g. flies of the genus Ormia; Ramsauer and Robert 1999).

The interactions of species with the human environment is also not yet properly covered. Roth and Willis (1960), for example, list the associations of various species of cockroach (Blattodea excluso Termitoidae) with buildings and vehicles (ships and aircraft). The Relations Ontology can handle this via abiotic-biotic interactions (http://www.ontobee.org/ontology/RO?iri=http://purl.obolibrary.org/obo/RO_0002446). Further work on the ecological interactions module would allow such occurrences to be recorded. Whether this is best dealt with using the methodology for inter-species interactions, with cockroaches interacting with the extended phenotype of humanity or, alternatively, these insects just being denizens of a highly mobile artificial microhabitat, is left for future discussion.

The data model we have developed is based upon the needs of the initial projects migrated and there is scope for future additions to accommodate additional needs (e.g. recording the date and time of observed interactions using DarwinCore eventTime).

There is great potential for the biodiversity informatics community to adopt or develop controlled vocabularies.

## Figures and Tables

**Table 1.** Organism status (presence status; aligned to DarwinCore establishmentMeans).

Status	Description
Native	The organism either evolved in this region or arrived by non-anthropogenic means.
Naturalised	The organism reproduces naturally and forms part of the local ecology.
Introduced	The organism arrived in the region via an anthropogenic mechanism or mechanisms.
Invasive	The organism is having a deleterious impact on another organism, multiple organisms or the ecosystem as a whole.
Captivity	The organism is kept in captivity.
Managed	The organism maintains its presence through intentional cultivation or husbandry.

**Table 2.** Wild/captive status of recorded interaction.

**Status**	**Description**
Interaction recorded in the wild	
Interaction recorded in captivity	Used when the status of the specimens is uncertain
Interaction recorded in captivity from wild caught specimens	
Interaction recorded in captivity from captive bred specimens	

**Table 3.** The importance to humans of ecological interactions.

**Status**	**Description**
Economic	The interaction has financial impact for human society
Economic (crop pest)	The interaction is damaging to food crops
Economic (timber pest)	The interaction is damaging to timber
Economic (pest control)	The interaction helps to control a pest species
Medical	The interaction has medical important consequences on humans
Veterinary	The interaction has medical important consequences on animals
